# Influence of cyclosporine and everolimus on the main mycophenolate mofetil pharmacokinetic parameters: Cross-sectional study

**DOI:** 10.1097/MD.0000000000006469

**Published:** 2017-03-31

**Authors:** Aurelija Noreikaitė, Franck Saint-Marcoux, Pierre Marquet, Edmundas Kaduševičius, Edgaras Stankevičius

**Affiliations:** a Institute of Physiology and Pharmacology, Medical Academy, Lithuanian University of Health Sciences; b INSERM UMR 850, Limoges; c Department of Pharmacology and Toxicology, CHU Limoges, Limoges Cedex; d University of Limoges, Limoges, France.

**Keywords:** cyclosporine, drug-to-drug interaction, mycophenolate mofetil, pharmacokinetics, renal transplantation

## Abstract

The objective of the present study was to assess the effect of cyclosporine (CsA) on the pharmacokinetic parameters of mycophenolic acid (MPA), an active mycophenolate mofetil (MMF) metabolite, and to compare with the effect of everolimus (EVR).

Anonymized medical records of 404 kidney recipients were reviewed. The main MPA pharmacokinetic parameters (AUC_(0–12)_ and C_max_) were evaluated.

The patients treated with a higher mean dose of CsA displayed higher MPA AUC_(0–12)_ exposure in the low-dose MMF group (1000 mg/day) (40.50 ± 10.97 vs 28.08 ± 11.03 h mg/L; *r*_s_ = 0.497, *P* < 0.05), medium-dose MMF group (2000 mg/day) (43.00 ± 6.27 vs 28.85 ± 11.08 h mg/L; *r*_s_ = 0.437, *P* < 0.01), and high-dose MMF group (3000 mg/day) (56.75 ± 16.78 vs 36.20 ± 3.70 h mg/L; *r*_s_ = 0.608, *P* < 0.05).

A positive correlation was also observed between the mean CsA dose and the MPA C_max_ in the low-dose MMF group (C_max_ 22.83 ± 10.82 vs 12.08 ± 5.59 mg/L; *r*_s_ = 0.507, *P* < 0.05) and in the medium-dose MMF group (22.77 ± 8.86 vs 13.00 ± 6.82 mg/L; *r*_s_ = 0.414, *P* < 0.01).

The comparative analysis between 2 treatment arms (MMF + CsA and MMF + EVR) showed that MPA AUC_(0–12)_ exposure was by 43% higher in the patients treated with a medium dose of MMF and EVR than in the patients treated with a medium dose of MMF and CsA.

The data of the present study suggest a possible CsA versus EVR influence on MMF pharmacokinetics. Study results show that CsA has an impact on the main MPA pharmacokinetic parameters (AUC_(0–12)_ and C_max_) in a CsA dose-related manner, while EVR mildly influence or does not affect MPA pharmacokinetic parameters. Low-dose CsA (lower than 180 mg/day) reduces MPA AUC_(0–12)_ exposure under the therapeutic window and may lead to ineffective therapy, while a high-dose CsA (>240 mg/day) is related to greater than 10 mg/L MPA C_max_ and increases the likelihood of adverse events.

## 1 Introduction

Immunosuppressive drugs are characterized by high variability in metabolism and pharmacokinetics that may result in drug toxicity or lack of efficacy.^[1]^ Chronic maintenance immunosuppression in transplantation requires special attention especially to the right dosage selection based on the assessment of plasma drug concentration. Low immunosuppressant concentration in plasma increases the risk of transplant rejection in the acute posttransplant period,^[2,3]^ while increased drug exposure may lead to the higher risk of adverse drug reactions,^[4,5]^ especially chronic allograft nephropathy.

The National Institute for Health and Care Excellence has outlined the recommendations for patients receiving kidney transplant.^[6]^ Basiliximab or daclizumab with or without cyclosporine (CsA) are recommended as an option for induction therapy. The National Institute for Health and Care Excellence has also noted that mycophenolate mofetil (MMF) should be used as an option as part of an immunosuppressive regimen only when intolerance to calcineurin inhibitors (CNIs), particularly nephrotoxicity leading to risk of chronic allograft dysfunction, is proven or in situations with a high risk of nephrotoxicity necessitating minimization or avoidance of a CNI.^[6]^ Meanwhile, the Kidney Disease Improving Global Outcomes Clinical Practice Guidelines recommend using a combination of a CNI and an antiproliferative agent with or without corticosteroids (CSs).^[7]^ However, in clinical practice, triple therapy with: a CNI (CsA); an antiproliferative agent (azathioprine or MMF); and a CS has been customarily constituted. Later on, many new regimens have been developed that incorporate rapid glucocorticoid elimination, CNI dose reduction or elimination due to numerous potential glucocorticoid, and CNI toxicities. CNI withdrawal has been attempted by conversion to less nephrotoxic mammalian target of rapamycin (mTOR) inhibitors.^[8–10]^ The MANDELA study (NCT00862979) initiated in 2009 was also designed to assess the benefit of either CNI-free or CNI-minimized everolimus (EVR)-based regimen.^[11]^

MMF is one of the components of triple therapy and an integral component of toxicity-sparing regimens that seek to minimize exposure to the nephrotoxic CNI.^[12]^ Recently, the need for guidelines on MMF dosing has increased as more individualized immunosuppressive drug regimens are used.^[13]^ However, MMF is of possible concern and its combination with drugs, environmental pollutants, or food constituents, which activate cytochrome P450 transcriptional factor, may represent a significant toxicological risk.^[14]^ An important detail related to the immunosuppressive regimen is that CsA used together with MMF inhibits the enterohepatic (re)circulation of mycophenolic acid (MPA), an active metabolite, and its inactive metabolite 7-O-glucuronide conjugate (MPAG) and results in significantly lower dose-corrected MPA concentrations in CsA-treated patients, which in turn will lead to early clinical MPA area under the concentration time curve (AUC) under exposure in 50%.^[15]^ The need to double the dose of MMF in case of CsA co-administration to achieve the same MPA levels have been emphasized,^[16]^ but is not always followed (usually in clinical practice the dose of MMF in co-administration of CsA is 2 g/day).

Genetic polymorphisms also play an important role. P-glycoprotein (Pgp) and cytochrome P450 3A4 have been recognized as determinants of the bioavailability of widely used immunosuppressants such as CsA, tacrolimus, and sirolimus. These immunosuppressants act as substrates and/or inhibitors of Pgp, alter the bioavailability of many concomitantly used drugs, and are potential inducers of drug–drug interactions.^[17]^

EVR, a derivative of sirolimus, is used in solid–organ transplantation and offers immunosuppression without CNI-induced toxicities.^[18,19]^ EVR in combination with MMF has shown promising renal outcomes after liver, heart, and kidney transplantation.^[20–22]^ Moreover, mTOR inhibitors have been shown to prevent tumors and even to reduce metastatic tumor growth by angiogenesis.^[23]^ Meanwhile, the combination of EVR and MMF used for immunosuppression has shown dose-dependent antiproliferative effects in tumor cell lines in vitro,^[24]^ and this is an additional benefit for the immunosuppression regimen, which in general poses a greater risk of cancer.^[6]^ These results strengthen the possibility of equilibrium between efficient immunosuppressive drug therapy and preservation over the development of cancer,^[25,26]^ thereby offering new therapeutic strategies for the treatment of malignancies in clinical practice.^[27,28]^ However, drug–drug interaction between these drugs might exist as well, and studies on the influence of EVR on MPA pharmacokinetic parameters are limited and it requires further evaluation.

The objective of the present study was to assess the influence of CsA on the main MPA pharmacokinetic parameters and to compare the effect of CsA and EVR on the main MPA pharmacokinetic parameters in patients with a renal graft.

## 2 Materials and methods

### 2.1 Characteristics of study patients

Anonymized medical records of 404 patients receiving immunosuppressant therapy after renal transplantation hospitalized at Limoges University Hospital (France) during the study period from 2011 to 2012 were reviewed. A total of 83 patients who received MMF and CsA therapy and 17 patients who received MMF and EVR therapy for approximately more than 1 year (17% of the patients received therapy for less than 1 year) were recruited and included in the study (Table 1). The inclusion criteria were age of more than 18 years, kidney transplant, and immunosuppression with either MMF and CsA therapy or MMF and EVR therapy. The patients were excluded if they received immunosuppression with other medicaments and underwent transplantation of other organs.

**Table 1:**
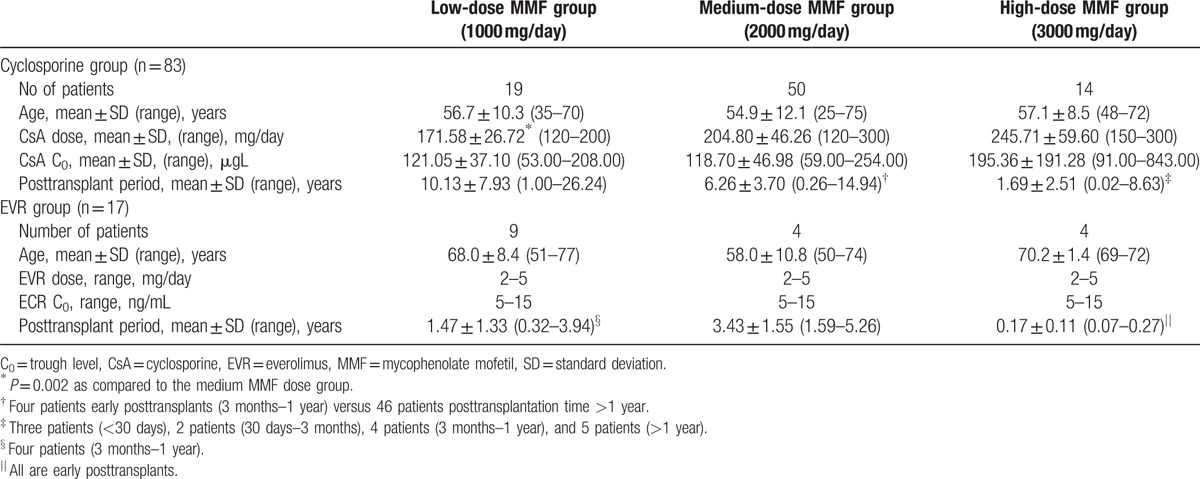
Characteristic of the study groups.

MMF and CsA were administrated twice daily, and EVR, once daily. The morning dose of EVR or CsA was given at the same time as that of MMF. All patients received prednisolone orally by standard hospital practice.

According to the MMF daily dose, the study patients were allocated into the 3 study groups: the low MMF dose group received 1000 mg per day (28 patients); medium MMF dose group, 2000 mg per day (54 patients); and high MMF dose group, 3000 mg per day (18 patients).

A CsA daily dose varied from 120 to 300 mg. CsA pharmacokinetic parameters during the study were maintained within the therapeutic window: trough level (C_0_) 132.2 ± 90.8 μg/L (therapeutic range of 75–150 μg/L for patients receiving long-term treatment); AUC_(0–12)_ 3.4 ± 0.9 h mg/L (dosage AUC 3.8 h mg/L); and maximal concentration (C_max_) 859.1 ± 253.0 μg/L. An EVR dose ranged from 2 to 5 mg/day, with target trough levels of 5 to 15 ng/mL. The characteristics of the study population are presented in Table 1. Protocol biopsies were performed and graded according to the Banff 97 classification.^[29]^

The study protocol was reviewed and approved by the Ethics Committee.

### 2.2 Determination of CsA

Blood samples were collected in EDTA tubes to measure the CsA C_0_ and drug-blood concentration 1 (C_1_) and 3 (C_3_) hours after the administration of CsA. CsA concentrations in the whole blood were measured using a validated turbulent-flow chromatography-tandem mass spectrometry technique.^[30]^ Online extraction was performed at 1.25 mL/min on a Cyclone P, 50 μm particle size (50 × 0.5 mm, id) column (Thermo Fisher) in alkaline conditions. Chromatographic separation was performed in acidic conditions (phase A 0.1% formic acid in water and phase B 0.1% formic acid in methanol) using a Propel MS C18, 5 μm (50 × 3.0 mm, id) column (Thermo Fisher) kept at 60 °C with a constant flow rate of 300 μL/min. Detection was performed using a TSQ Quantum Discovery tandem mass spectrometer equipped with an orthogonal electro spray ionization source and controlled by the XCalibur software (Thermo Fisher). Tandem mass spectrometry was performed in the positive ion multiple reaction monitoring (MRM) mode following 3 transitions for CsA (m/z 1220.0 → 1203.0 for quantification and m/z 1220.0 → 1185.0 and m/z 1220.0 → 425.0 for confirmation) and 2 transitions (m/z 1234.0 → 1217.0 for quantification and m/z 1234.0 → 119.0 for confirmation) for its analogue CsA D, used as an internal standard (IS). Methanol/aqueous zinc sulfate (200 μL, 70:30 v/v) containing the internal standard at 25 μg/L was added to the whole blood (100 μL). The mixture was vortexed for 45 seconds and centrifuged at 13,000 rpm, and the supernatant was introduced into a 200-μL vial for injection. Calibration standards at 0, 10, 20, 50, 100, 200, 500, 1000, and 2000 μg/L of CsA were prepared by spiking blank blood. The limits of detection (LOD) and quantification (LOQ) were 10 and 20 μg/L, respectively, and calibration curves obtained using quadratic regression from the LOQ to 2000 μg/L yielded *r*^2^ > 0.99.

### 2.3 Determination of MPA

Blood samples were collected in EDTA tubes at 20 minutes, 1 and 3 hours after the administration of MMF. Plasma was separated by centrifugation. The measurement of total MPA was performed using a validated high-performance liquid chromatography (HPLC) method with ultraviolet (UV) detection.^[31]^ Blood serum (500 μL), an internal standard (50 μL) (thiopental in methanol 1 g/L diluted with deproteinized water to 25 mg/L), and calibrators were acidified with hydrochloric acid and extracted with dichloromethane (5 mL). Calibrators were prepared in drug-free plasma and their concentrations were 0, 0.5, 1, 5, 10, and 20 μg/L for MPA. The organic fraction was then evaporated to dryness under a stream of nitrogen. The dry residue was reconstituted with 100-μL elution solvent (KH_2_PO_4_ buffer/acetonitrile [70/30 v/v] at pH = 2.6). Then, the sample (40 μL) was injected into the HPLC system with a steel column Nucleosil C18, 5 μm (250 × 4.6 mm, id) and with UV detection at 300 nm. The limits of LOD and LOQ were 50 and 200 μg/L, respectively, and calibration curves obtained using quadratic regression from the LOQ to 20,000 μg/L yielded *r*^2^ > 0.999.

### 2.4 Pharmacokinetic analysis

The NONMEM version VI (GloboMax LLC) nonlinear mixed-effects population pharmacokinetic model and the Bayesian estimator of a 3-point limited sampling strategy developed at Limoges University Hospital were used to determine CsA^[32,33]^ and MPA^[34]^ area under the blood concentration–time curve (AUC_(0–12)_).

### 2.5 Statistical analysis

The values of MPA pharmacokinetic parameters (AUC_(0–12)_ and C_max_) were compared between the patients’ groups receiving dual therapy with MMF and CsA (doses ranged from 120 to 300 mg/day), and between the patients of 3 treatment arms receiving MMF and CsA versus patients receiving MMF and EVR. IBM SPSS 20.0 was used for statistical analysis. Probability values of less than 0.05 were considered significant. Correlation coefficients were calculated using the Spearman and Pearson correlation tests. Brian P O’Connor Parallel Analysis (PA) to for determining the number of components to retain from principal components analysis (PCA) component was used on SPSS. PCA eigenvalues from the data greater than PA eigenvalues from the corresponding random data were retained. All components with eigenvalues below this threshold value were considered spurious.^[35]^ The unpaired *t* test was used to compare the study groups (GraphPad software, available online: http://www.graphpad.com/quickcalcs/ttest1.cfm). The relationship between MPA AUC_(0–12)_, CsA AUC_(0–12)_, and dose was assessed with a linear regression analysis model.

## 3 Results

### 3.1 Analysis of CsA influence on MPA

A large interindividual variation of MPA pharmacokinetic data was observed with different MMF doses from 1000 to 3000 mg/day within each group (receiving dual therapy with MMF and CsA). A significant positive correlation within the MMF groups was noticed between the main MPA pharmacokinetic parameters (AUC_(0–12)_ and C_max_). The patients treated with a higher CsA dose (180–240 mg/day) displayed higher MPA AUC_(0–12)_ exposure than those who were treated with a low CsA dose (120–180 mg/day) in the low-dose MMF group (1000 mg/day) (40.50 ± 10.97 vs 28.08 ± 11.03 h mg/L; *r*_s_ = 0.497, *P* < 0.05), medium-dose MMF group (2000 mg/day) (43.00 ± 6.27 vs 28.85 ± 11.08 h mg/L; *r*_s_ = 0.437, *P* < 0.01); and high-dose MMF group (3000 mg/day) (56.75 ± 16.78 vs 36.20 ± 3.70 h mg/L; *r*_s_ = 0.608, *P* < 0.05).

The same positive correlation was also observed between a CsA dose and MPA C_max_. The patients treated with a high CsA dose (180–240 mg/day) had increased C_max_ compared with the patients treated with a low CsA dose (120–180 mg/day) in the low-dose MMF group (1000 mg/day) (22.83 ± 10.82 vs 12.08 ± 5.59 mg/L) and in the medium-dose MMF group (2000 mg/day) (22.77 ± 8.86 vs 13.00 ± 6.82 mg/L). Spearman correlation coefficients were *r*_s_ = 0.507 (*P* < 0.05) and *r*_s_ = 0.414 (*P* < 0.01) in the low- (1000 mg/day) and medium-dose MMF groups, respectively. The comparison of pharmacokinetic parameters between the patients’ groups is demonstrated in Table 2.

**Table 2:**
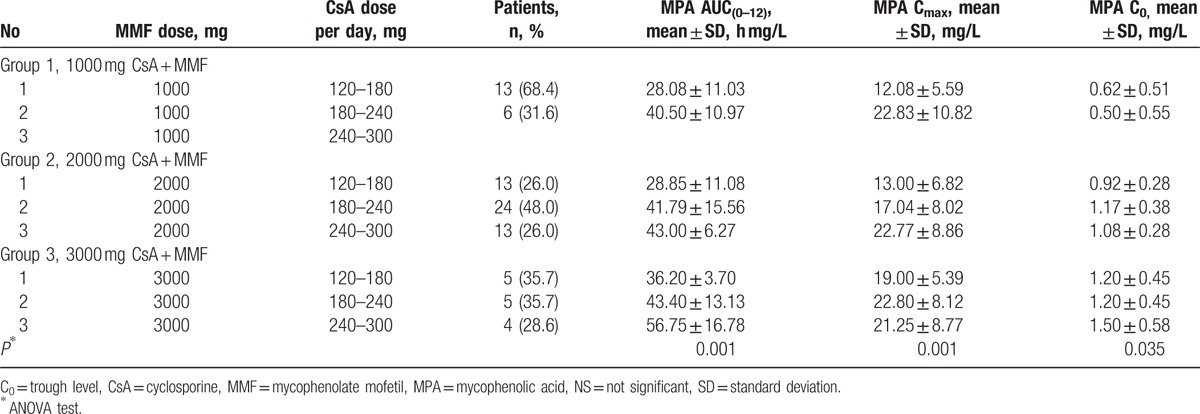
Comparison of pharmacokinetic parameters between the study groups.

**Figure 1:**
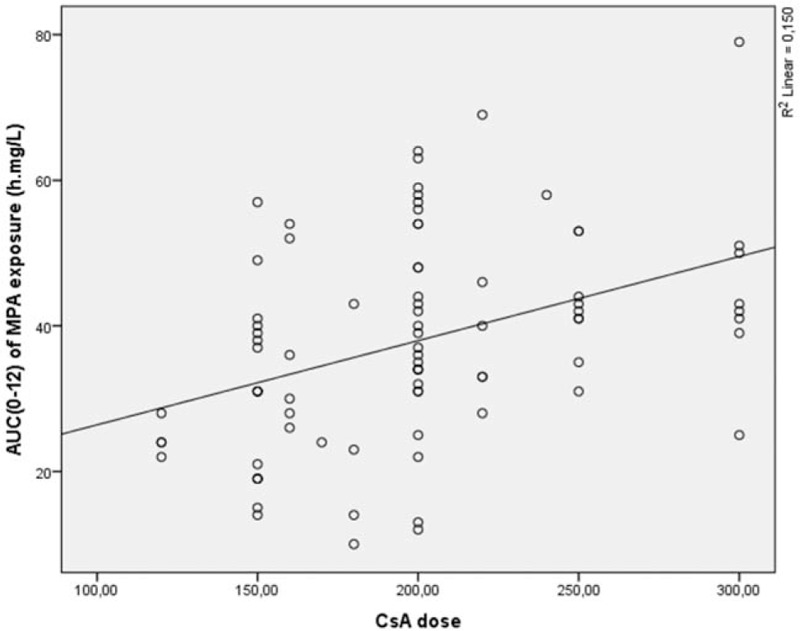
AUC_(0–12)_ of MPA exposure dependence from CsA dose. AUC = area under the concentration time curve, CsA = cyclosporine, MPA = mycophenolic acid.

**Figure 2:**
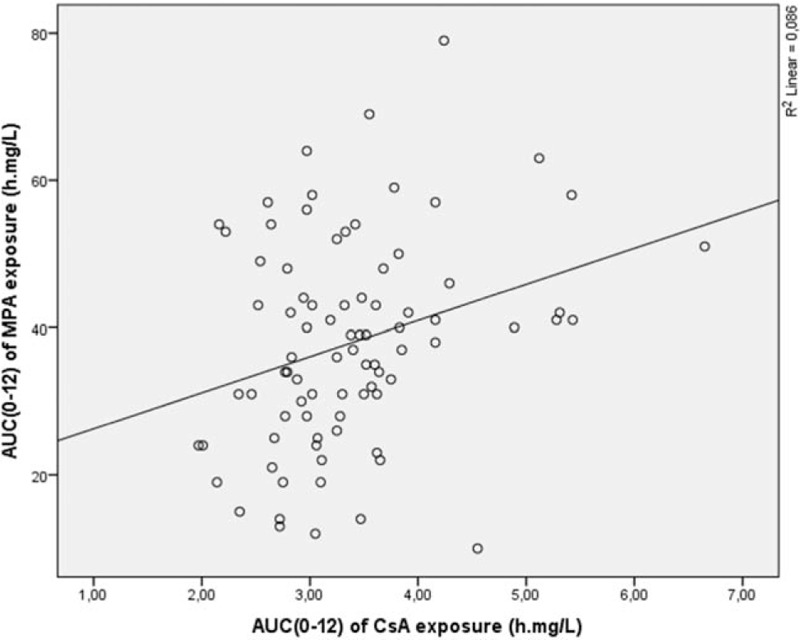
AUC_(0–12)_ of MPA exposure dependence from CsA AUC_(0–12)_ exposure. AUC = area under the concentration time curve, CsA = cyclosporine, MPA = mycophenolic acid.

**Figure 3:**
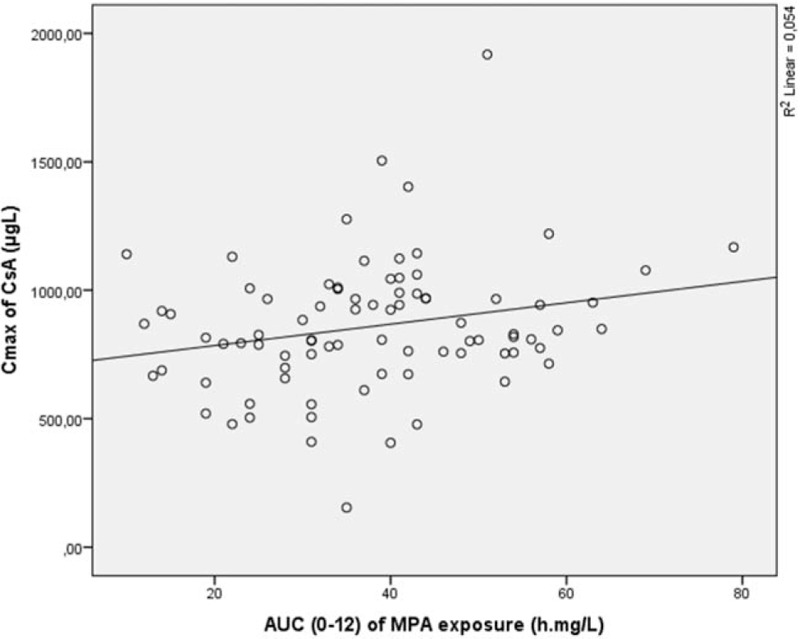
C_max_ of CsA dependence from AUC_(0–12)_ of MPA exposure. AUC = area under the concentration time curve, C_max_ = maximal concentration, CsA = cyclosporine, MPA = mycophenolic acid.

For the full-scale data analysis linear regression was performed. Analysis showed that the AUC_(0–12)_ of MPA was CsA dose dependent and accounted 15.0% of the cases (*r* = 0.385, *P* < 0.01) (Fig. 1). Moreover, weak dependency was noticed between the AUC_(0–12)_ of MPA and CsA AUC_(0–12)_, and this dependency explained only 8.6% of the cases (*r* = 0.293, *P* < 0.01) (Fig. 2). The AUC_(0–12)_ of MPA dependency on CsA C_max_ explained 5.4% of the cases (*r* = 0.232, *P* < 0.05) (Fig. 3).

**Figure 4:**
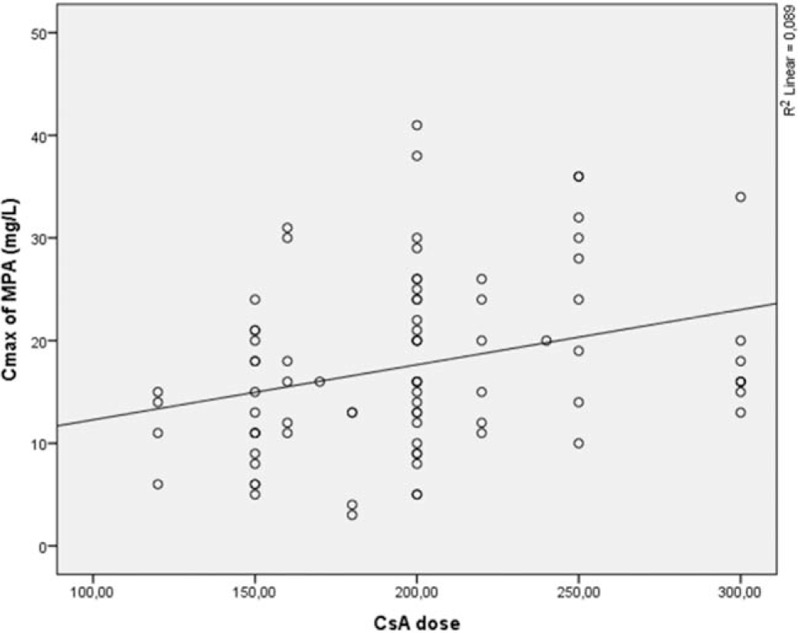
C_max_ of MPA dependence from CsA dose. C_max_ = maximal concentration, CsA = cyclosporine, MPA = mycophenolic acid.

**Figure 5:**
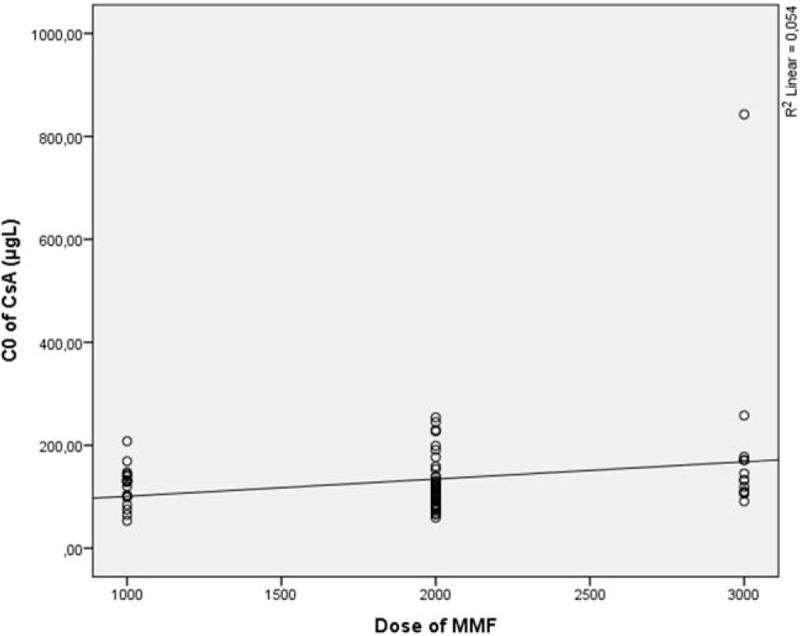
C_0_ of CsA dependence from MMF dose. C_0_ = trough level, CsA = cyclosporine, MMF = mycophenolate mofetil.

MPA C_max_ significantly correlated with a CsA dose (*r* = 0.299, *P* < 0.01) (Fig. 4), and MPA C_0_ significantly correlated with CsA AUC_(0–12)_ (*r* = 0.296, *P* < 0.01). No correlation was observed between CsA C_0_ and MPA pharmacokinetic parameters, but an MMF dose significantly correlated with CsA C_0_ (*r*_s_ = 0.221 (*P* < 0.05) (Fig. 5). Such drug-to-drug interaction and MPA AUC exposure dependency on CsA dose, CsA AUC, and CsA C_max_ as well as MPA C_max_ dependency on CsA dose and MPA C_0_ dependency on CsA AUC showed a strong relationship between CsA and MPA what might play a key role in individual therapy.

### 3.2 Use of parallel analysis

Parallel analysis was performed using 3 components and 5 variables: CsA dose, CsA C_0_, CsA AUC_(0–12)_ exposure value, MPA C_0_, MPA AUC_(0–12)_ exposure value. The results of the parallel analysis test showed that there was only 1 component to be retained for interpretation. A CsA dose should be retained and considered as the only 1 factor affecting the MMF AUC exposure.

### 3.3 Manifestation of chronic allograft nephropathy

In CsA co-administration groups, chronic allograft nephropathy (classification of MEDRA 18.0) was diagnosed in 36.8% of the patients in the low MMF dose group (7 of 19 patients), in 24.0% of the patients in the medium MMF dose group (12 of the 50 patients), and in 7.1% of the patients in the high MMF dose group (1 of the 14 patients). The presence of chronic allograft nephropathy did not correlate with MPA AUC exposure, but negatively correlated with MPA C_0_ (*r* = –0.262, *P* = 0.017) when MMF was co-administrated with CsA.

In EVR co-administration groups, chronic allograft nephropathy was diagnosed in 77.8% of the patients in the low MMF dose group (7 of the 9 patients), in 50.0% of the patients in the medium MMF dose group (2 of the 4 patients), and in 50.0% of the patients in the high MMF dose group (2 of the 4 patients). In total, chronic allograft nephropathy was diagnosed in 64.7% of the patients (11 of the 18 patients). There was a negative moderate correlation between the presence of chronic allograft nephropathy and MPA AUC exposure when MMF was co-administrated with EVR (*r* = –0.508, *P* = 0.037).

### 3.4 Comparison of CsA and EVR effect on the main MPA pharmacokinetic parameters

The comparative analysis between 2 treatment arms (CsA + MMF vs EVR + MMF) showed a statistically significant difference in pharmacokinetic parameters. MPA C_0_ and MPA AUC_(0–12)_ were significantly lower in the CsA + MMF treatment arm compared with the EVR + MMF treatment arm (Tables 3 and 4). The greatest difference in the MPA C_0_ and MPA AUC_(0–12)_ between the CsA + MMF and EVR + MMF treatment arms was observed in the medium MMF dose (2000 mg) group (1.08 ± 0.34 vs 4.17 ± 0.78 mg/L and 38.74 ± 13.74 vs 68.69 ± 22.68 mg/L, respectively). In the patients’ group, where a medium MMF dose (2000 mg) was co-administered with EVR, the MPA AUC_(0–12)_ was by 43% higher than in the patients’ group where a medium MMF dose (2000 mg) was co-administered with CsA.

**Table 3:**
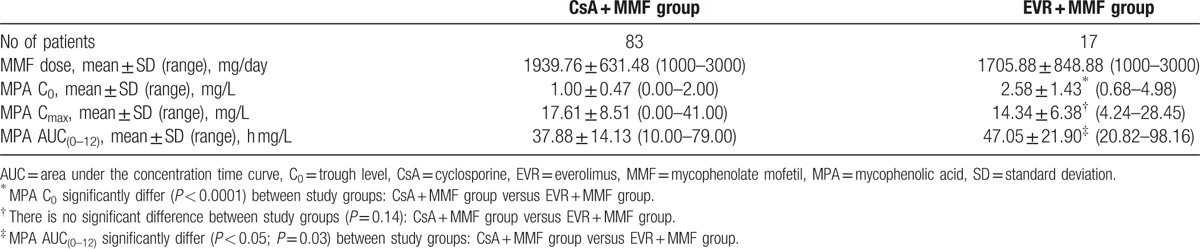
Comparison of MMF pharmacokinetic parameters between CsA + MMF and EVR + MMF study groups.

**Table 4:**
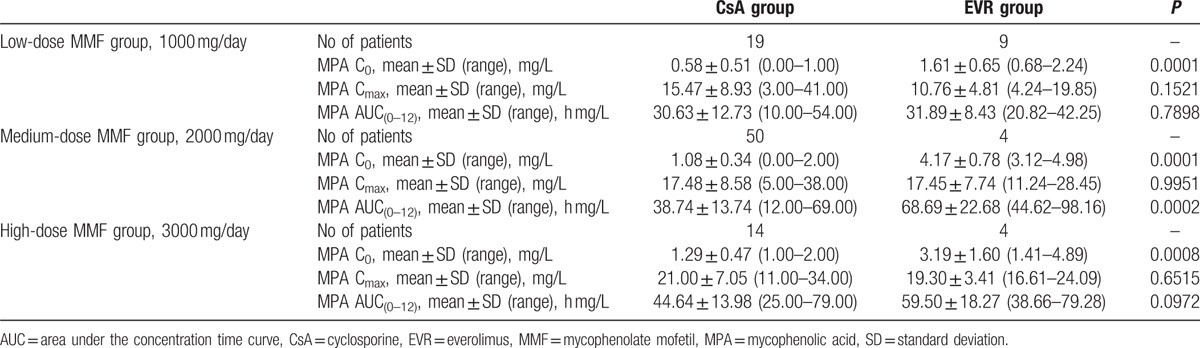
Comparison of pharmacokinetic parameters between study subgroups.

## 4 Discussion

The results obtained in our study show the influence of CsA and EVR on the MPA plasma level. So far, the impact of sirolimus on the MPA plasma level has been investigated,^[36,37]^ and MPA trough levels higher than expected have been documented by Cattaneo et al.^[38]^ This is in agreement with the findings of our study where the MPA AUC was by 43% higher in the MMF + EVR than the MMF + CsA group.

Variation of MPA pharmacokinetic parameters between the study groups possibly is the evidence of CsA and MMF drug-to-drug interaction, which has been noticed by other studies as well.^[15,39,40]^ Grinyo et al^[12]^ studied the influence of standard- and low-dose CsA on MPA exposure and found that CsA reduced the MPA exposure. It was documented by comparing CsA groups with low-dose tacrolimus or low-dose sirolimus groups. Filler and Feber^[41]^ analyzed immunosuppressant interactions, including drug interactions between CsA and tacrolimus with MPA, in renal transplant children and concluded that different MMF doses were required with either CsA or tacrolimus to obtain the same results.

Several different mechanisms of drug interactions in order to explain the relation between MPA and CsA have been proposed by researchers, although the ultimate mechanism has not been elucidated yet. The most likely mechanism by which CsA reduces MPA enterohepatic recirculation is through inhibition of the multidrug resistance-associated protein 2 (MRP2) transporter; however, other, not yet identified, canalicular transporters might be implied.^[42–44]^

It is thought that CsA interacts with the enterohepatic cycling of MPA by inhibiting the MRP2.^[42]^ MPAG biliary excretion decreases because of MRP2 inhibition caused by CsA. This leads to a diminution of MPA intestinal reabsorption (after deconjugation by the intestinal flora) and reduction in recirculation of MPA. MPAG displaces MPA from its protein binding sites, leading to an increased unbound fraction of MPA. If only the MPA–MPAG metabolic pathway is inhibited by CsA, an MPA increase must be linked to a decrease in MPAG.

The role of the MRP2 transporter in the hepatic disposition of MPA and MPAG has been studied widely in animal models.^[42–45]^ Animal studies have demonstrated that CsA, but not tacrolimus, plays a role in inhibiting the biliary excretion of MPAG by the MRP2 transporter^[42,45,46]^ and is the mechanism responsible for the interaction between CsA and MMF. Nevertheless, the clinical importance of the model approved on animals remains unanswered. Tetsuka et al^[47]^ have made it more complicated. They hypothesized that the sinusoidal efflux of MPA and/or MPAG was affected by CsA. In their study on sandwich-cultured hepatocytes, MPAG reduction by CsA was found. The authors identified that acyl-glucuronide, a minor MPA metabolite, did not change the biliary excretion index, which suggests that unique or additional transporter(s) are involved in biliary excretion of acyl-glucuronide. However, experimental evidence that CsA decreased the enterohepatic recirculation of MPA shortly after transplantation has been confirmed by Cattaneo et al in humans.^[38]^ We also demonstrated this tendency despite dose lowering required for long-term treatment.

Some researchers believe that MMF may interact with mTOR inhibitors. Proliferation signal inhibitors such as sirolimus and EVR are substrates of cytochrome P450 3A4 and Pgp and have a macrolide structure very similar to tacrolimus, which explains why common drug interactions with proliferation signal inhibitors are comparable to those with CNIs.^[48]^ Another important observation is that the MPA AUC differs not only between the groups, but also within the groups. A significant difference was observed not only between different MMF doses, but also between different CsA doses. The MPA AUC was approximately 33% lower in the low CsA dose group (120–180 mg) versus the high CsA dose group (240–300 mg). That leads us to think that MRP2 inhibition in the gastrointestinal track might not be the only mechanism related to low MPA AUC exposure. The bioavailability and metabolism of CsA are controlled by efflux pumps belonging to the ABC transporter family as Pgp and members of the cytochrome P-450 isoenzyme, and CsA can thus be involved in the activity of efflux pumps. Pgp system activity on CsA bioavailability might delay or disturb absorption that can introduce large variability in drug response or alter the bioavailability of concomitant drugs. It has been shown that in the patients with a CT or CC nucleotide exchange (high pumpers) in exon 26 (C3435T) with high Pgp activity on the apical surface of intestinal enterocytes, more CsA is removed from the cells, which results in decreased bioavailability.^[49]^ In this context, patients (high pumpers) treated with low doses of CsA would show lower drug exposure and this could affect MPA AUC exposure.^[17,49]^

In addition, a significant difference in the MPA C_max_ suggests that the initial absorption of MMF is also CsA or MMF dose dependent (*r*_s_ = 0.507, *P* < 0.05 and *r*_s_ = 0.414, *P* < 0.01, respectively) (Table 2). These findings support the importance of therapeutic drug monitoring when designing combined immunosuppressive regimens.

In scientific discussion for the approval of CellCept, it has been noted that despite clearly defined data about the CsA effect on MPA levels, the interaction between CellCept and CsA has no clinical implication and current monitoring instructions are satisfactory.^[50]^ Although this is a clear statement, researchers still insist that the use of a combination of drugs (CsA plus MMF) requires therapeutic drug monitoring and this study is not an exception. Better outcomes are achieved when therapeutic drug blood monitoring is performed.^[51]^ In the present study, the MPA AUC was outside the therapeutic window range (range 30–60 h mg/L) in 34% of the study patients (31.3% in MMF + CsA vs 47.00% in MMF + EVR), and fewer cases of chronic allograft nephropathy were noted with a high MMF dose (3000 mg/day) (1.2% in MMF + CsA vs 11.8% in MMF + EVR). It was proved that each 1 h mg/L increase in the MPA area under the plasma concentration (not exceeding AUC exposure range 30–60 h mg/L) versus time curve was associated with a 4% decreased risk of an event such as acute rejection, graft loss, or death (HR = 0.96; 95% CI: 0.93–0.99). This means that the higher MPA AUC might not induce chronic allograft nephropathy and MMF dosing relatively safely can be increased if MMF is co-administrated with CsA in order to achieve the same MPA AUC.^[52]^

Prescription of CsA and MMF is still important while such CNI withdrawal therapies as EVR combination with MMF are more expensive^[53]^ and not available in low-income countries. However, MMF + EVR therapy is more advanced than CsA + MMF therapy taking into account CsA-induced nephrotoxicity and other adverse effects.

## 5 Conclusions

The data of the present study suggest a possible CsA versus EVR influence on MMF pharmacokinetics. Study results show that CsA has an impact on the main MPA pharmacokinetic parameters (AUC_(0–12)_ and C_max_) in a CsA dose-related manner, while EVR mildly influences or does not affect MPA pharmacokinetic parameters. Low-dose CsA (lower than 180 mg/day) reduces MPA AUC_(0–12)_ exposure under the therapeutic window and may lead to ineffective therapy, while a high-dose CsA (>240 mg/day) is related to greater than 10 mg/L MPA C_max_ and increases the likelihood of adverse events.

## 6 Limitations

This study involved a small number of patients, and more accurate data can be obtained in larger study groups. The high-dose MMF group included 14 patients: 5 early posttransplantation time patients versus 9 moderate posttransplantation time patients. The results of this group are limited. Moreover, all the study patients received glucocorticoid treatment in combination with MMF and CsA therapy according to hospital guidelines, but the influence of glucocorticoid use on the main MMF and CsA pharmacokinetic parameters was not evaluated.
